# Pleuropulmonary blastoma type I might arise in congenital pulmonary airway malformation type 4 by acquiring a Dicer 1 mutation

**DOI:** 10.1007/s00428-020-02789-6

**Published:** 2020-03-19

**Authors:** Luka Brcic, Fabian Fakler, Sylvia Eidenhammer, Andrea Thueringer, Karl Kashofer, Janina Kulka, Helmut Popper

**Affiliations:** 1 Diagnostic and Research Institute of Pathology, Medical University of Graz, Neue Stiftingtalstrasse 6, 8036 Graz, Austria; 2 Second Department of Pathology, Semmelweis University Budapest, Budapest, Hungary

**Keywords:** Congenital pulmonary airway malformation, CPAM 4, Dicer 1 mutation, Pleuropulmonary blastoma, RAS family oncogenes

## Abstract

Congenital pulmonary airway malformation (CPAM) occurs most commonly in infants. It is divided into 5 types. The most common types 1 and 2 are cystic, type 0 presents as bronchial buds without alveolar tissue, most likely corresponding to alveolar dysgenesis, while type 3 is composed of branching bronchioles and appears as a solid lesion. A defect in the epithelial-mesenchymal crosstalk might be the underlying mechanism for all. Type 4 is a peripheral cystic lesion with a thin cyst wall covered by pneumocytes. CPAM 4 has been mixed up with pleuropulmonary blastoma (PPB) type I and some authors question its existence. We investigated five cases of CPAM type 4 for the presence or absence of rhabdomyoblasts, and for markers associated with CPAM development. In addition, all cases were evaluated for mutations within the Dicer gene and for mutations of the RAS family of oncogenes. All five cases showed smooth muscle actin and desmin-positive cells; however, only one case showed a few cells positive for MyoD. The same case showed a mutation of Dicer 1. All cases were negative for mutations of the RAS family of genes. Fibroblast growth factor 10 was similarly expressed in all cases, and thus cannot be used to differentiate CPAM4 from PPB-I. Low expression of the proliferation marker Ki67 was seen in our CPAM 4 cases and the probable PPB-I case. YingYang-1 protein seems to play an active role in the development of PPB-I. CPAM 4 can be separated from PPB-I based on the presence of rhabdomyoblasts and mutations in Dicer 1 gene. These cells might not be numerous; therefore, all available tissue has to be evaluated. As CPAM 4 morphologically looks very similar to PPB-I, it might be speculated, that there exists a potential for progression from CPAM 4 to PPB-I, by acquiring somatic mutations in Dicer 1.

## Introduction

Congenital pulmonary airway malformation (CPAM) is a rare disease of the lung. The incidence rate is between 1:11000 and 1:35000 in newborns [1, 2]. Due to the ongoing improvements in prenatal ultrasound technology, most of the cystic malformations are detected intrauterine [3, 4]. This might explain, why the incidence of CPAM continues to rise. CPAM, formerly known as congenital cystic adenomatoid malformation (CCAM), was described in 1949 as a distinct entity by Ch’in and Tang [5]. The Stocker classification divided it initially into types 1, 2, and 3 [6]. In 2002, Stocker modified the classification by adding two new types, 0 and 4, and changed the name from congenital cystic adenomatoid malformation to congenital pulmonary airway malformation [7]. This change was made due to the fact that not all subtypes are cystic, and only one of them is adenomatoid.

CPAM type 4 is a peripheral cystic lesion, defined as a defective growth of distal alveoli. It is rarer than the types 1–3. In CPAM 4, the cysts are covered by an alveolar type epithelium, which can be flat or cuboidal (transformed pneumocytes type II). No connection with bronchial or bronchiolar structures is present. The stroma of CPAM 4 has not been thoroughly investigated [8–11]. Histologically, it looks very similar to pleuropulmonary blastoma type I (PPB), another lung disease predominantly affecting young children. Three types of PPB are discerned, all of them can occur in the lung, in the pleura, or in both. PPB types II and III are morphologically quite characteristic due to the occurrence of solid structures with the cambium layer and many rhabdomyoblasts and chondrosarcoma islands especially in the latter. Type I being entirely cystic [12, 13] and has often be mixed-up with CPAM 4. In earlier reports, CPAM 4 was published as a pleuropulmonary blastoma occurring in CPAM or associated with it [14, 15]. Even Stocker, in his classification, mentioned problems in separating CPAM 4 from PPB-I. He characterized CPAM 4 as a cystic lesion with an epithelial layer of pneumocytes type I and II, and a cyst wall, which is thin in younger, and thicker in older patients, but still loosely populated by stroma cells. In his update, Stocker more precisely mentioned the absence of rhabdomyoblasts in CPAM 4 [16]. The epithelial layer in PPB-I was said to be cuboidal or columnar, and the stroma consists of a cambium layer. In addition, PPB should have chromosomal abnormalities such as trisomy 2 and 8 [7].

We aimed to characterize CPAM 4 by immunohistochemistry, to analyze mutations of Dicer 1 and RAS family genes, and to compare those with the findings in PPB-I.

## Methods

### Patient samples

The archive of the Diagnostic and Research Institute of Pathology, Medical University of Graz, Graz, Austria, was searched for the following diagnostic terms: “CCAM,” “CPAM,” “Sequestration,” “Alveolar Adenoma,” and “Pleuropulmonary Blastoma.” All retrieved cases were sectioned again, stained with hematoxylin-eosin, and reviewed by two pathologists (HP, LB) in order to confirm the diagnosis. One case of CPAM 4 was contributed by one coauthor. Basic clinical data were obtained for all patients (Table 1).

**Table 1:** Age distribution of CPAM 4 at the time of surgery

	Sex	Age (years)	Recurrence
Case 1	Female	62	No
Case 2	Male	1	No
Case 3	Male	17	No
Case 4	Female	2	No
Case 5	Male	19	No

The study protocol was approved by the local University Ethics Committee (Number 24-135 ex 11/12).

### Immunohistochemistry

Four-micrometer thick sections were prepared from all available paraffin blocks and incubated with antibodies for smooth muscle actin (SMA), desmin (DES), and MyoD, to characterize smooth muscle cells, myofibroblasts, and rhabdomyoblasts. Pan-cytokeratin antibodies (CK) were used to characterize the surface epithelium. S100 protein was included to detect neurogenic cells, and Ki67 to characterize the proliferation capacity. As an association of fibroblast growth factor 10 (FGF10) with CPAM formation has been published previously, FGF10 was included in this study [17, 18]. YingYang 1 protein (YY1) involved in protein-DNA, protein-RNA, and protein-protein interactions, and regulating developmental processes [19, 20] was also included. Detailed immunohistochemical protocols are presented in Table 2.

**Table 2:** List of antibodies and techniques of immunohistochemistry; *r.t.u.*, ready to use

Name, clone	Company	Dilution	Pretreatment	Detection
SMA	Sigma	1:5000	CC1	iView DAB Ventana
DES	Dako	r.t.u.	EnV Flex TRS high pH Omnis	Flex HRP DAB Omnis
FGF10	Abcam	1:1000	MW9,0 (S2367 DAKO)	ENV DAB (K5007 DAKO)
CK	Dako	1:100	Protease 1	iView DAB Ventana
S100	Dako	1:2000	Protease 2	iView DAB Ventana
Ki67	Dako	r.t.u	EnV Flex TRS low pH Omnis	Flex HRP DAB Omnis
YY1	Abcam	1:500	CC1	ultraView DAB Ventana
MyoD	Dako	1:50	MW6,0 (S1699 DAKO)	ENV DAB (K5007 DAKO)

### Next generation sequencing

All five cases were investigated for mutations of Dicer 1 gene and with a lung cancer panel including RAS gene family members. The CPAM cysts were marked and macrodissected from several sequential sections. In addition, tissue was also macrodissected from normal lung, adjacent to the cysts. DNA was extracted using the Maxwell RSC DNA FFPE Kit (Promega, Mannheim, Germany) and quantified with Picogreen on a Qbit fluorometer (Life Tech Austria, Vienna, Austria). Next generation sequencing (NGS) libraries were prepared using the AmpliSeq library kit 2.0 (Thermo Fisher Scientific) and the Ion Ampliseq Cancer Hotspot Panel V2 (CatNr: 4475346) primer pool covering hotspot mutations in 50 genes implicated in cancer and a custom Ampliseq Panel covering all exons of Dicer 1. Sequencing was performed on an Ion Proton benchtop sequencer (Thermo Fisher Scientific) to a length of 200 base pairs. Initial data analysis was done using the Ion Torrent Suite Software Plug-ins (Thermo Fisher Scientific, open source, GPL, https://github.com/iontorrent/). Briefly, this included base calling, alignment to the reference genome (HG19) using the TMAP mapper, and variant calling by a modified diBayes approach considering the flow space information. Called variants were annotated using open source software ANNOVAR [21] and SnpEff [22]. All coding, nonsynonymous mutations were further evaluated and visually inspected in IGV (http://www.broadinstitute.org/igv/) and variant calls resulting from technical read errors or sequence effects were excluded from the analysis.

## Results

**Fig. 1:**
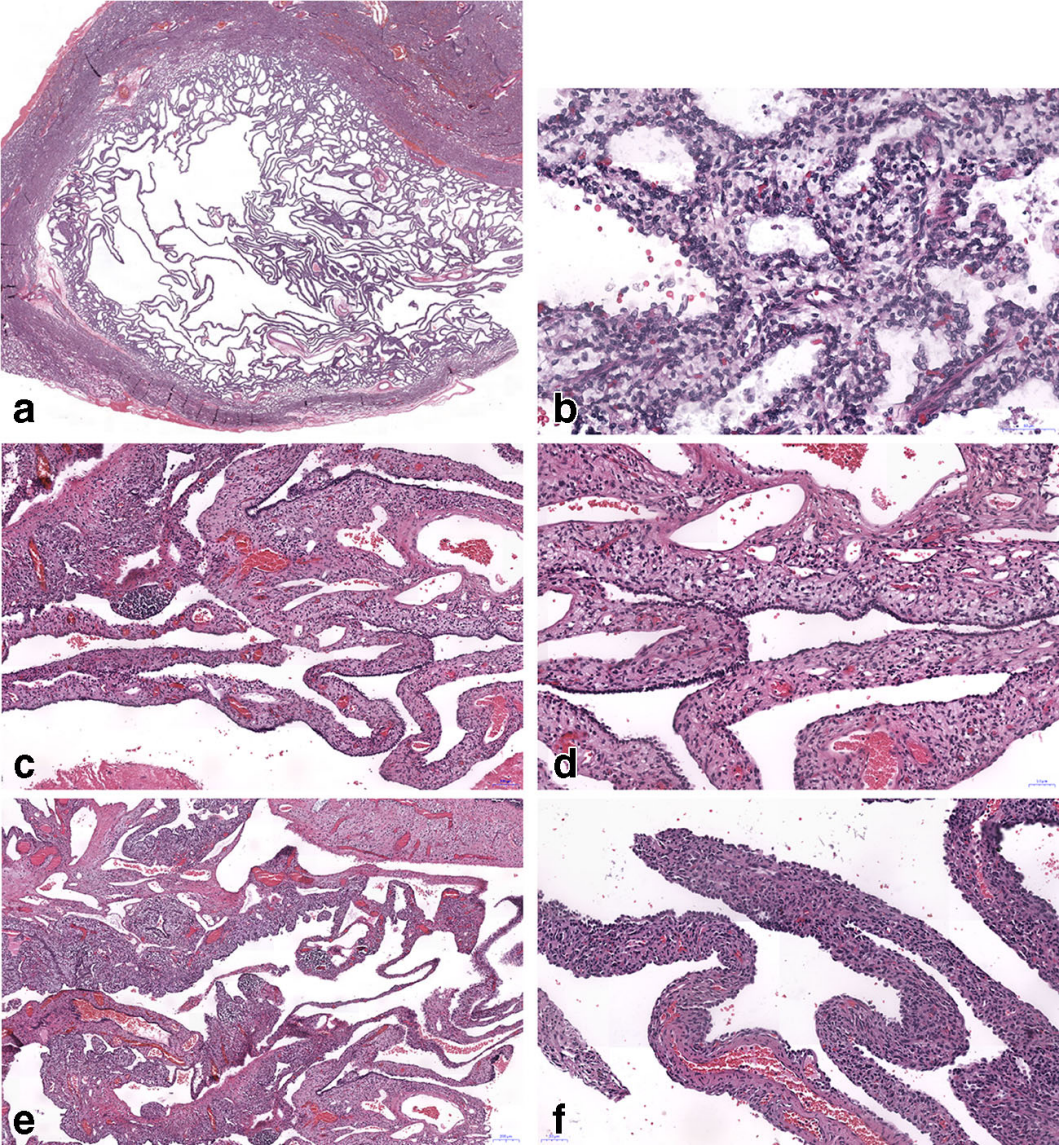
**a**–**e** Three CPAM 4 cases are illustrated; the case in **a** showed a mutation in the DICER 1 gene, whereas **c** and **d** are negative being confirmed as CPAM 4; **b**, **d**, and **f** show corresponding higher magnification of the cases_,_ H&E, bars 100, and 50 μm, respectively

**Fig. 2:**
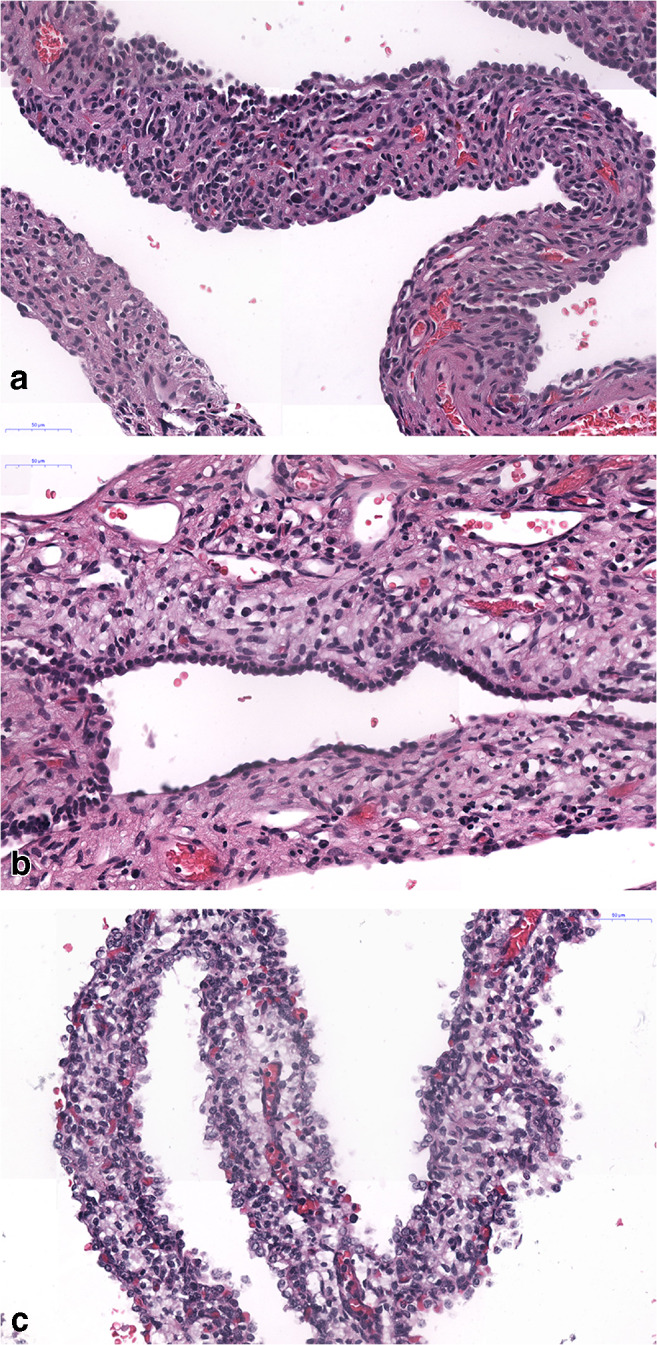
High magnification of CPAM 4 cases, **a** and **b** are negative and **c** being positive for Dicer 1 mutation. In this area, the cellularity is similar in all three cases. H&E, bars 50 μm

A total of 19 cases of CPAM, 2 cases of pleuropulmonary blastoma type III, and one referral case of PPB II were identified from the archive. After reevaluation, four cases of CPAM 4 were identified (including cases from consultation files), and a fifth case of CPAM 4 was contributed by another author, giving a total of 5 cases for investigation. All CPAM 4 cases fulfilled the Stocker criteria: multiple peripheral cysts covered by an alveolar type surface epithelium (pan-cytokeratin positive), cyst walls with thin to medium stroma thickness (Fig. 1). The mesenchymal cells were composed of primitive cells with round or ovoid nuclei with dense chromatin and invisible nucleoli. The cytoplasm was eosinophilic, in some cases finely vacuolated, cell borders could not be discerned. A few scattered larger cells presented with round slightly enlarged nuclei and small round nucleoli. The chromatin in these cells was finely granular. Lymphocytic infiltrations were focally present. Focally smooth muscle cells and myofibroblasts could be identified with elongated nuclei and eosinophilic cytoplasm with structures corresponding most probably to myofilaments. In one case, there were focal regressive changes with fibrosis, but without necrosis. On high power magnification, large scattered cells were seen in stroma, suspicious for rhabdomyoblasts (Figs. 1 and 2).

### Immunohistochemistry

**Fig. 3:**
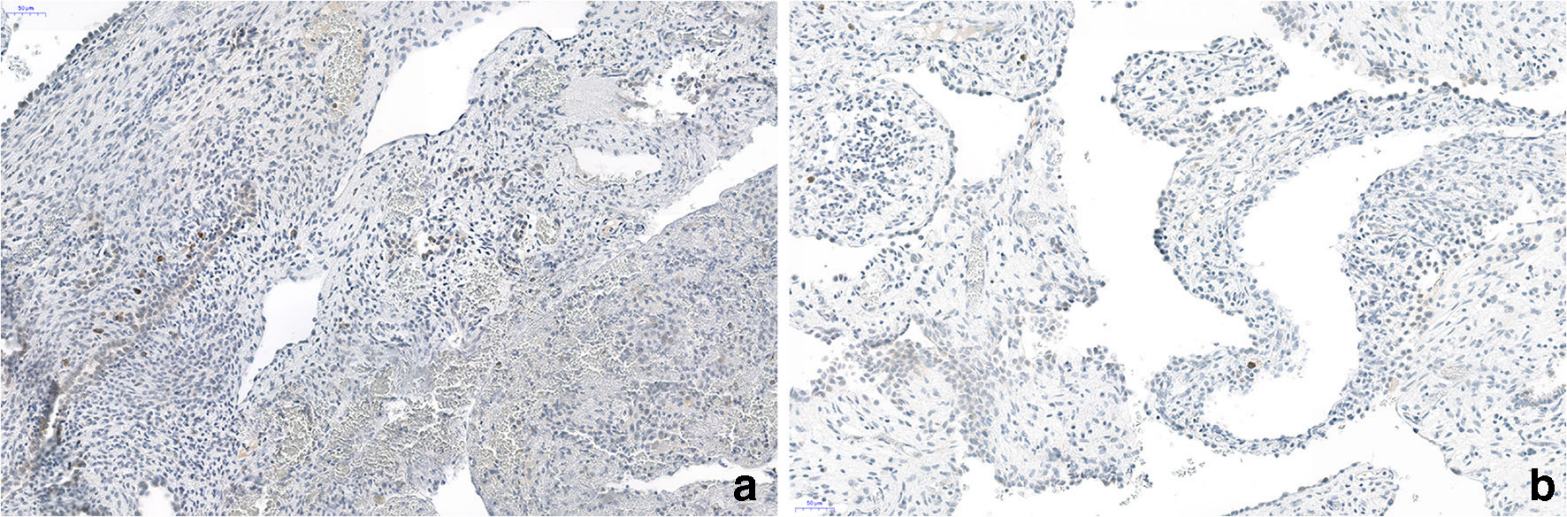
Immunohistochemistry for MyoD. In **a**, the case with Dicer 1 mutation, in **b**, a case with no positive cells. Note the focal group of positive cells (nuclear staining), other areas were negative. Some cells in **b** showed an unspecific cytoplasmic staining. Bars 50 μm

**Fig. 4:**
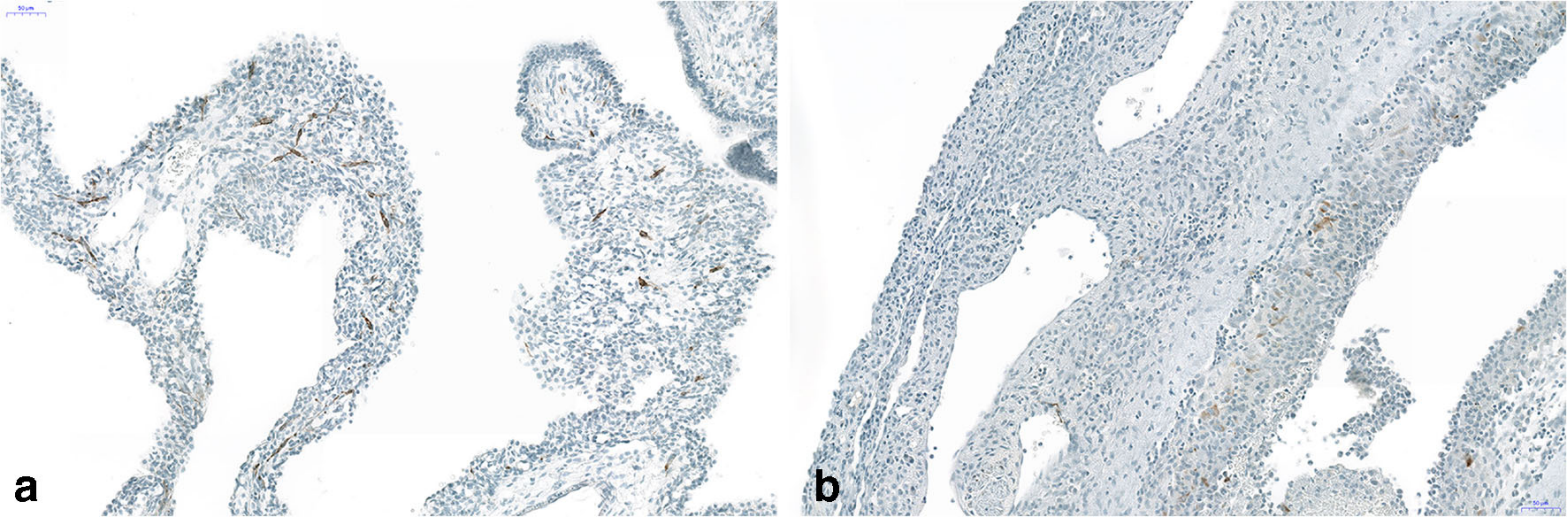
Immunohistochemistry for desmin. Both cases show a few scattered cells, stained with this antibody. **a** Case with Dicer 1 mutation. **b** Negative for Dicer 1. Bars 50 μm

**Fig. 5:**
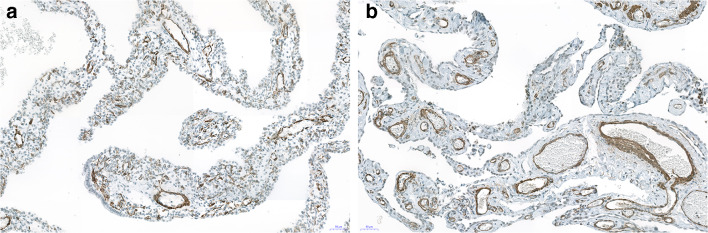
**a**, **b** Immunohistochemistry for smooth muscle actin showing positively stained stroma cells corresponding to myofibroblasts; some mature smooth muscle cells with more intense staining can be seen in blood vessel walls. Of note is that many stroma cells are negative, probably more primitive mesenchymal precursor cells. Bars 50 μm

Single desmin and MyoD-positive rhabdomyoblasts could be identified in 1 of 5 cases, whereas desmin-positive single cells were also seen in 3 other cases, being negative for MyoD (Figs. 3 and 4). In 3 cases, we could investigate several tissue blocks, but MyoD-positive cells were not detected. A continuous layer of rhabdomyoblasts and primitive fetal-type stroma cells (so-called cambium layer) was not seen in any of the cases, although few of the cells with vacuolated cytoplasm might represent some primitive mesenchymal cells. In all five cases, the interstitium was focally thickened and most often composed of myofibroblasts, positively stained for SMA (Fig. 5).). However, SMA-positive myofibroblasts were not encountered in every area, it seems that primitive mesenchymal cells only focally differentiated into myofibroblasts (Fig. 5).

**Fig. 6:**
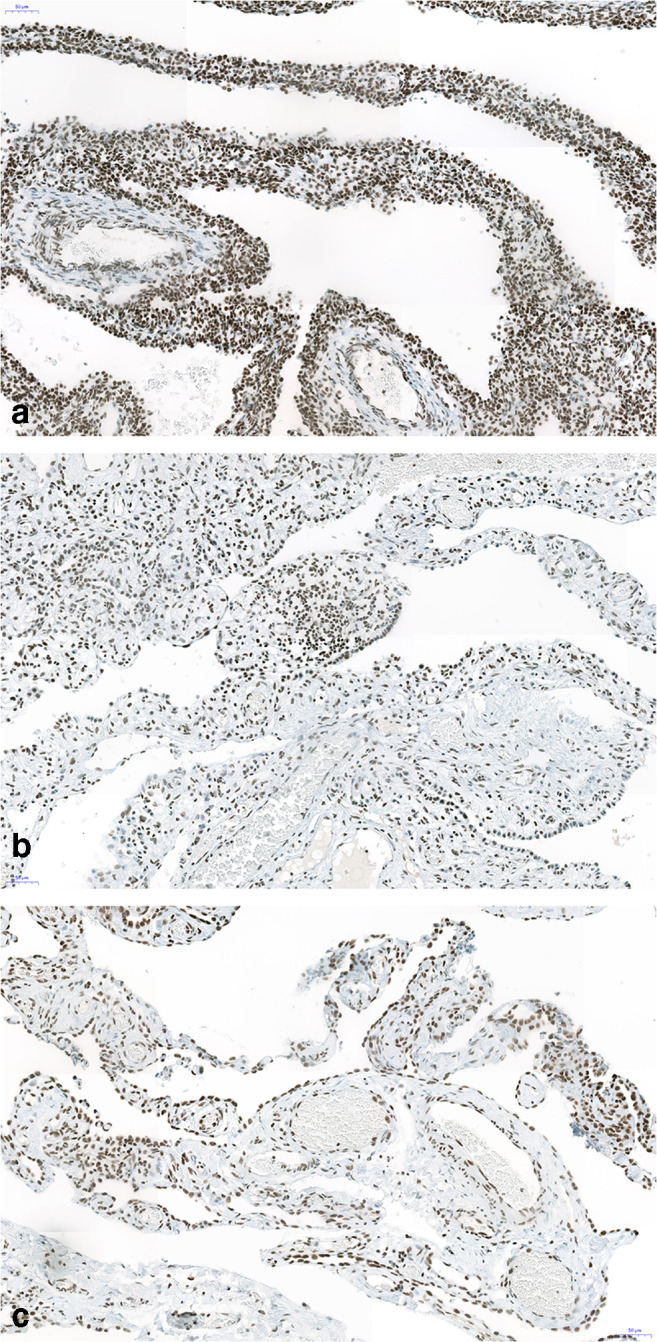
Strong nuclear expression of YY1 protein in the epithelium as well as in almost all stroma cells. In **a**, there seem to be a higher cellularity and more intense staining (case with Dicer 1 mutation), whereas both other cases (**b**, **c**) are less intensely stained and present with less stroma cells. Bars 50 μm

**Fig. 7:**
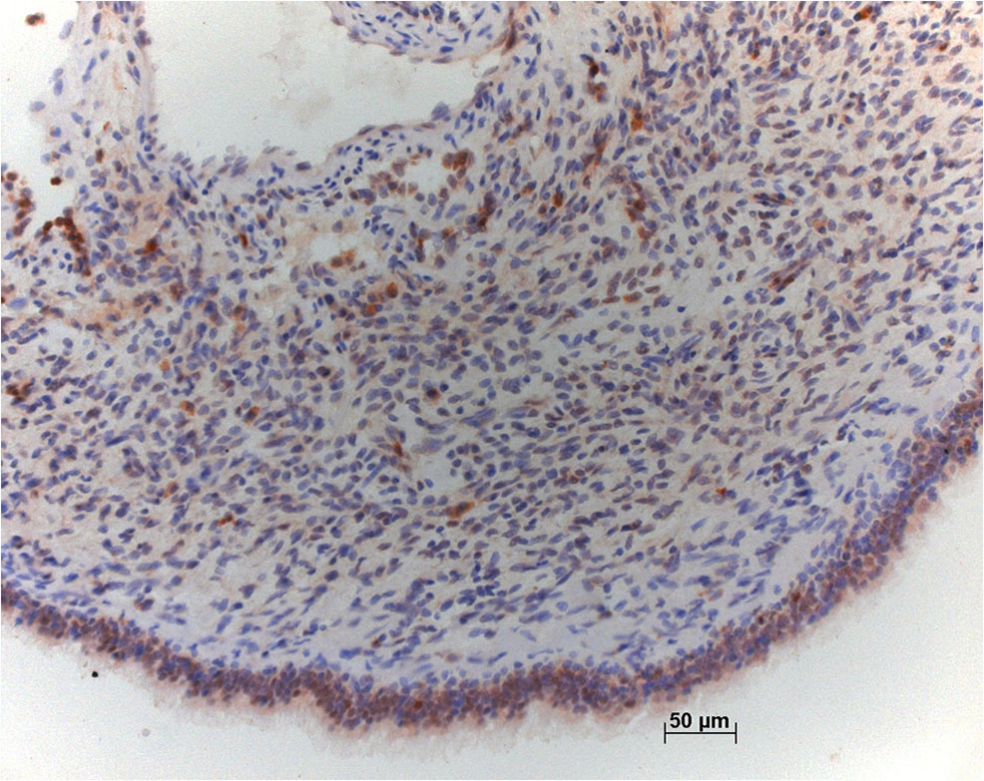
Staining for FGF10 shows intense staining in the epithelium covering this case with Dicer 1 mutation. Some stroma cells are positively stained too, including questionable rhabdomyoblasts. Bar 50 μm

FGF10 was positive in the surface epithelium in all cases, whereas the stroma cells were focally positive in 4 of the 5 cases (Fig. 6). YY1 was expressed focally in the nuclei of the epithelium and in the stroma cells in all cases, the staining intensity being higher in the epithelium compared with the stroma (Fig. 7). A strong expression was also seen in presumable rhabdomyoblasts and in general in the Dicer 1 mutation-positive case (Fig. 7). Ki67 was rarely positive in the nuclei of stroma cells, in general ≤ 1%, often confined to the questionable rhabdomyoblasts. S100 protein was negative in all cases.

By NGS, no mutations within the RAS family genes were found (KRAS; NRAS; HRAS, BRAF). In one case initially diagnosed as CPAM 4, a Dicer 1 mutation was found (NM_001271282:exon25:c.G5437A:p.E1813K, (hg19,chr14:95557630,C/T) MAF: 24.12 und 19.27%, Sift Score), whereas no mutation could be seen in the four other cases.

## Discussion

We investigated five cases, which have been diagnosed as CPAM type 4 based on the published criteria of multiple large peripheral cysts covered by pneumocytes and thin-walled stroma. Typical primitive mesenchymal cells never formed a continuous layer (cambium layer), as it is seen in classical PPB-I. There were focal primitive appearing mesenchymal cells, similar to what can be seen in other diseases, such as pulmonary interstitial glycogenosis, but negative for glycogen. Based on the finding of few desmin-positive cells, also MyoD immunohistochemistry was applied, which labeled few cells in only one case. This case with MyoD-positive cells had a Dicer 1 mutation, exactly in the hot spot region for pleuropulmonary blastomas. This raises the question, if CPAM type 4 can be differentiated from PPB-I, and if CPAM 4 can progress into PPB-I?

In his initial report, Stocker defined CPAM 4 as multicystic peripheral lung lesion covered by pneumocytes [7]. He stated that the main differences between PPB-I and CPAM 4 are a cuboidal epithelium in PPB, a thicker mesenchyme with a cambium layer, and bronchial epithelium at the edges. Even Stocker raised doubts, if CPAM 4 can be differentiated from PPB-I: “I have seen one case of PPB presented as a ‘CPAM’ that was partially resected at age 2 only to recur as a solid nodule of PPB at age 4.” In a recent report he added that the presence of desmin-positive rhabdomyoblasts rules out CPAM 4 [16]. Looking at published figures of CPAM 4, they all showed thin-walled cysts and thin-walled stroma. However, as Stocker added, the thickness of the cyst wall can increase in older patients, probably due to inflammation, cyst rupture, and subsequent repair.

In our CPAM 4 cases, we analyzed the surface epithelium as well as the stroma. As PPB can occur in the lung, pleura, or both, the covering cells might be bronchiolar, alveolar, or mesothelial; therefore, in pleural tumors, the epithelium might be flat, similar to pneumocytes type I [23, 24]. However, these cells are not part of the tumor. In CPAM 4, the surface cells have been characterized as pneumocytes, often positively stained for TTF1. And these cells are part of the CPAM lesion. However, most important is the analysis of the stroma. The stroma in PPB-I is composed of rhabdomyoblasts, primitive fetal cells, and myofibroblasts. In CPAM 4, there are loose stroma cells, some primitive mesenchymal cells, myofibroblasts but no rhabdomyoblasts [7, 16]. When comparing our cases, the similarity is obvious: the surface epithelium is flat or cuboidal, the stroma is slightly thickened in all; some cases show inflammation with lymphocytes and features of repair. Since none of our cases had a layer of rhabdomyoblasts underneath the epithelium, nor a cambium-layer of primitive mesenchymal cells, a HE-based diagnosis of PPB-I was impossible. The appearance of desmin-positive cells raised the question, if all cases might be labeled as PPB-I. However, desmin is not entirely specific for rhabdomyoblasts, as other cells can be stained too. MyoD staining reduced the suspected cases to only one. This resulted in the investigation for mutations in all cases.

Different immunohistochemical markers have also been investigated for the differentiation between PPB-I and CPAM 4. Downregulation of FGF10 and YY1 in PPB-I has been proposed to assist in the separation from CPAM 4. However, our case of probable PPB-I arising in CPAM 4 showed the same staining pattern as the other 4 cases, only the expression of YY1 was more intense compared with the other cases. Therefore, in our opinion, this might not be helpful in all cases. As we do not have more cases of PPB-I, a further comparison was impossible—and PPB-II and PPB-III do not help in this respect.

The confusion between PPB I and CPAM 4 came from earlier reports. CPAM 4 has been described before the Stocker classification as CPAM associated with pleuropulmonary blastoma or rhabdomyosarcoma arising in CPAM [14, 15]. Some authors have even raised the question if CPAM 4 exists [11, 23–28]. In the report by Sweeney, CPAM 4 showed stromal hypercellularity, and a PPB I developed subsequently in this patient. In the report by Vargas, the incidence of TP53 mutations and numerical aberrations in chromosome 8 was investigated in CPAM and PPB-I [29]. The authors concluded that CPAM is non-neoplastic, because only 2 of 10 cases showed TP53 mutations, but no chromosomal abnormalities. It is important that in this study the authors selected CPAM cases without any subtyping, so most likely they have included other CPAM types, such as the more common types 1 and 2. Their statement that CPAM, as well as PPB-I, “show benign epithelium-lined cysts and mesenchymal proliferation” is misleading: Whereas in CPAM 1–4, the epithelium is part of the lesion, in PPB I-III, the pulmonary and pleural epithelium covers the malignant tumor and is not part of the tumor. In some cases, labeled as regressive, the malignant mesenchymal phenotype might be missing, which complicates this issue [23].

In his initial description, Stocker noticed that the main difference between CPAM 4 and PPB-I is the cambium layer in PPB. However, a continuous cambium layer is seen in PPB II and III, whereas in PPB-I, there are small subepithelial rows or only scattered rhabdomyoblasts within the mesenchymal stroma of the cyst wall. In addition, the primitive stroma cells, easily identified in PPB-II and PPB-III, cannot always be recognized in PPB-I. In our limited experience with PPB, the most important feature is a cell-rich mesenchymal stroma, present in our mutated case, which should prompt one to stain for smooth muscle actin, desmin, and MyoD, which seems to be more specific. In addition, a mutation analysis for Dicer 1 should be done to better separate it from CPAM, although not all cases of PPB I will show this mutation [30]. Other mutations have been also demonstrated in PPB such as NRAS and BRAF, but were absent in our case [31].

In a recent controversy on this subject, Dehner et al. [32] argued that CPAM 4 is identical to PPB-I, and that there is a possibility that these lesions evolve from pure cystic into partially cystic-semisolid and finally into the solid type III PPB. In response, Lamas-Pinheiro et al. [33] argued that YY1 and FGF10 are differently expressed in CPAM and PPB I (downregulated in PPB). However, we did not see a downregulation of YY1 and FGF10 in the stroma or epithelium in our mutated case, when compared with our CPAM 4 cases.

Another question is, if our cases are all PPB-I? In a much larger series of PPB, Dicer 1 mutation was not seen in all cases, however, regressive changes in those cases being negative [23]. In our cases, we neither have seen necrosis nor extensive fibrosis. The stroma cells all look actively proliferating, although the proliferation index was very low, consistent with a low-grade lesion.

Based on our limited experience, we think CPAM 4 does exist, but we propose that all cases of peripheral cystic lesions should be primarily evaluated for rhabdomyoblasts within the stroma. In addition, a mutation analysis for Dicer 1 and RAS gene family members should be done. Our case with Dicer 1 mutation supports the hypothesis already discussed in previous reports that stroma cells within CPAM 4 might acquire a somatic Dicer 1 mutation and progress into PPB-I.

## References

[1] Durell J, Lakhoo K (2014). Congenital cystic lesions of the lung. Early Hum Dev.

[2] Laberge JM, Flageole H, Pugash D, Khalife S, Blair G, Filiatrault D, Russo P, Lees G, Wilson RD (2001). Outcome of the prenatally diagnosed congenital cystic adenomatoid lung malformation: a Canadian experience. Fetal Diagn Ther.

[3] Gornall AS, Budd JLS, Draper ES, Konje JC, Kurinczuk JJ (2003). Congenital cystic adenomatoid malformation: accuracy of prenatal diagnosis, prevalence and outcome in a general population. Prenat Diagn.

[4] Stocker LJ, Wellesley DG, Stanton MP, Parasuraman R, Howe DT (2015). The increasing incidence of foetal echogenic congenital lung malformations: an observational study. Prenat Diagn.

[5] Ch’In KY, Tang MY (1949). Congenital adenomatoid malformation of one lobe of a lung with general anasarca. Arch Pathol.

[6] Stocker JT, Madewell JE, Drake RM (1977). Congenital cystic adenomatoid malformation of the lung. Classification and morphologic spectrum. Hum Pathol.

[7] Stocker JT (2002). Congenital pulmonary airway malformation: a new name and an expanded classification of congenital cystic adenomatoid malformation of the lung. Histopathol.

[8] Bush A (2001). Congenital lung disease: a plea for clear thinking and clear nomenclature. Pediatr Pulmonol.

[9] Morotti RA, Cangiarella J, Gutierrez MC, Jagirdar J, Askin F, Singh G, Profitt SA, Wert SE, Whitsett JA, Greco MA (1999). Congenital cystic adenomatoid malformation of the lung (CCAM): evaluation of the cellular components. Hum Pathol.

[10] Ortac R, Diniz G, Yildirim HT, Aktas S, Karaca I (2016). Retrospective evaluation of children with congenital pulmonary airway malformation: a single center experience of 20 years. Fetal Pediatr Pathol.

[11] van Koningsbruggen S, Ahrens F, Brockmann M, Michalk D, Rietschel E (2001). Congenital cystic adenomatoid malformation type 4. Pediatr Pulmonol.

[12] Dehner LP (1994). Pleuropulmonary blastoma is THE pulmonary blastoma of childhood. Semin Diagn Pathol.

[13] Manivel JC, Priest JR, Watterson J, Steiner M, Woods WG, Wick MR, Dehner LP (1988). Pleuropulmonary blastoma. The so-called pulmonary blastoma of childhood. Cancer.

[14] Ohno M, Takezoe T, Watanabe T, Tahara K, Hishiki T, Fujino A, Matsuo M, Higuchi M, Kawasaki K, Shioda Y, Kato M, Kiyotani C, Matsumoto K, Takakuwa E, Irie R, Yoshioka T, Kimura S, Seki M, Takita J, Kanamori Y (2017) A female case of pleuropulmonary blastoma type 1 whose pulmonary cystic lesion was followed since neonate, 34-37

[15] Thakkar HS, Durell J, Chakraborty S, Tingle BL, Choi A, Fowler DJ, Gould SJ, Impey L, Lakhoo K (2017). Antenatally detected congenital pulmonary airway malformations: the Oxford experience. Eur J Pediatr Surg.

[16] Stocker JT (2009). Cystic lung disease in infants and children. Fetal Pediatr Pathol.

[17] Gonzaga S, Henriques-Coelho T, Davey M, Zoltick PW, Leite-Moreira AF, Correia-Pinto J, Flake AW (2008). Cystic adenomatoid malformations are induced by localized FGF10 overexpression in fetal rat lung. Am J Res Cell Mol Biol.

[18] Lezmi G, Verkarre V, Khen-Dunlop N, Vibhushan S, Hadchouel A, Rambaud C, Copin MC, Rittie JL, Benachi A, Fournet JC, Delacourt C (2013). FGF10 signaling differences between type I pleuropulmonary blastoma and congenital cystic adenomatoid malformation. Orphanet J Rare Dis.

[19] Boucherat O, Landry-Truchon K, Berube-Simard FA, Houde N, Beuret L, Lezmi G, Foulkes WD, Delacourt C, Charron J, Jeannotte L (2015). Epithelial inactivation of Yy1 abrogates lung branching morphogenesis. Development.

[20] Dinglin X, Ding L, Li Q, Liu Y, Zhang J, Yao H (2017). RYBP inhibits progression and metastasis of lung cancer by suppressing EGFR signaling and epithelial-mesenchymal transition. Transl Oncol.

[21] Wang K, Li M, Hakonarson H (2010). ANNOVAR: functional annotation of genetic variants from high-throughput sequencing data. Nucleic Acids Res.

[22] Cingolani P, Platts A, Wang L, Coon M, Nguyen T, Wang L (2012). A program for annotating and predicting the effects of single nucleotide polymorphisms. SnpEff: SNPs in the genome of *Drosophila melanogaster* strain w1118; iso-2; iso-3. Fly (Austin).

[23] Hill DA, Jarzembowski JA, Priest JR, Williams G, Schoettler P, Dehner LP (2008). Type I pleuropulmonary blastoma: pathology and biology study of 51 cases from the international pleuropulmonary blastoma registry. Am J Surg Pathol.

[24] Priest JR, Williams GM, Hill DA, Dehner LP, Jaffe A (2009). Pulmonary cysts in early childhood and the risk of malignancy. Pediatr Pulmonol.

[25] d’Agostino S, Bonoldi E, Dante S, Meli S, Cappellari F, Musi L (1997). Embryonal rhabdomyosarcoma of the lung arising in cystic adenomatoid malformation: case report and review of the literature. J Pediatr Surg.

[26] Feinberg A, Hall NJ, Williams GM, Schultz KA, Miniati D, Hill DA, Dehner LP, Messinger YH, Langer JC (2016). Can congenital pulmonary airway malformation be distinguished from type I pleuropulmonary blastoma based on clinical and radiological features?. J Pediatr Surg.

[27] MacSweeney F, Papagiannopoulos K, Goldstraw P, Sheppard MN, Corrin B, Nicholson AG (2003). An assessment of the expanded classification of congenital cystic adenomatoid malformations and their relationship to malignant transformation. Am J Surg Pathol.

[28] Nasr A, Himidan S, Pastor AC, Taylor G, Kim PC (2010). Is congenital cystic adenomatoid malformation a premalignant lesion for pleuropulmonary blastoma?. J Pediatr Surg.

[29] Vargas SO, Korpershoek E, Kozakewich HP, de Krijger RR, Fletcher JA, Perez-Atayde AR (2006). Cytogenetic and p53 profiles in congenital cystic adenomatoid malformation: insights into its relationship with pleuropulmonary blastoma. Pediatr Dev Pathol.

[30] Slade I, Bacchelli C, Davies H, Murray A, Abbaszadeh F, Hanks S, Barfoot R, Burke A, Chisholm J, Hewitt M, Jenkinson H, King D, Morland B, Pizer B, Prescott K, Saggar A, Side L, Traunecker H, Vaidya S, Ward P, Futreal PA, Vujanic G, Nicholson AG, Sebire N, Turnbull C, Priest JR, Pritchard-Jones K, Houlston R, Stiller C, Stratton MR, Douglas J, Rahman N (2011). DICER1 syndrome: clarifying the diagnosis, clinical features and management implications of a pleiotropic tumour predisposition syndrome. J Med Genet.

[31] Pugh TJ, Yu W, Yang J, Field AL, Ambrogio L, Carter SL, Cibulskis K, Giannikopoulos P, Kiezun A, Kim J, McKenna A, Nickerson E, Getz G, Hoffher S, Messinger YH, Dehner LP, Roberts CW, Rodriguez-Galindo C, Williams GM, Rossi CT, Meyerson M, Hill DA (2014). Exome sequencing of pleuropulmonary blastoma reveals frequent biallelic loss of TP53 and two hits in DICER1 resulting in retention of 5p-derived miRNA hairpin loop sequences. Oncogene.

[32] Dehner LP, Messinger YH, Williams GM, Stewart DR, Harney LA, Schultz KA, Hill DA (2017). Type I pleuropulmonary blastoma versus congenital pulmonary airway malformation type IV. Neonatology.

[33] Lamas-Pinheiro R, David M, Henriques-Coelho T (2017). Reply to the Letter to the Editor ‘Type I Pleuropulmonary Blastoma versus Congenital Pulmonary Airway Malformation Type IV’. Neonatology.

